# Protective effects of mangafodipir against chemotherapy-induced ovarian damage in mice

**DOI:** 10.1186/s12958-018-0426-y

**Published:** 2018-10-27

**Authors:** Ying Qin, Akira Iwase, Tomohiko Murase, Bayasula, Chiharu Ishida, Nao Kato, Tomoko Nakamura, Satoko Osuka, Sachiko Takikawa, Maki Goto, Tomomi Kotani, Fumitaka Kikkawa

**Affiliations:** 1 Department of Obstetrics and Gynecology, Nagoya University Graduate School of Medicine, 65 Tsurumai-cho, Showa-ku, Nagoya, 466-8550 Japan; 2 Department of Obstetrics and Gynecology, Gunma University Graduate School of Medicine, 3-39-22 Showa-machi, Maebashi, 371-8511 Japan; 3 Department of Maternal and Perinatal Medicine, Nagoya University Hospital, 65 Tsurumai-cho, Showa-ku, Nagoya, 466-8550 Japan

**Keywords:** Anticancer drug, Follicle, Mangafodipir, Ovary, Oxidative stress

## Abstract

**Background:**

Given the seriousness of chemotherapy-induced ovarian injury in female cancer patients, the preservation of fertility, including through the use of cryopreservation technology and pharmaceuticals, requires investigation. Previous studies have shown that damage to the ovaries is related to oxidative stress caused by anticancer drugs. Therefore, superoxide dismutase (SOD) may represent a key factor in the pharmacological protection of the ovaries. The aim of our study was to identify the effects of mangafodipir, a manganese chelate and SOD-mimetic, on suppression of apoptosis in granulosa cells and primordial follicle activation induced by anticancer drugs.

**Methods:**

Cell viability assays using methyltrichlorosilane solutions and immunoblotting for cleaved caspase-3 were performed in in vitro experiments with the simultaneous addition of mangafodipir to human non-luteinized granulosa cell line (HGrC) cultures treated with hydrogen peroxide (H_2_O_2_), cisplatin, or paclitaxel. Count and morphological analyses of follicles at each developing stage in the ovaries and immunohistochemistry for cleaved caspase-3, Ki67 and 4-hydroxynonenal, a marker for oxidative stress, were also performed using mangafodipir-injected 6-week-old female ICR mice treated with cisplatin or paclitaxel. Further, mangafodipir was injected into 6-week-old female BALB/c mice inoculated with ES-2 to analyze whether mangafodipir inhibits the anti-tumor effects of cisplatin or paclitaxel treatment.

**Results:**

Mangafodipir attenuated apoptosis induced by H_2_O_2_ and anticancer drugs in vitro. Mangafodipir also decreased the expression of 4-hydroxynonenal and reduced cisplatin- and paclitaxel-induced apoptosis in granulosa cells in vivo. In addition, mangafodipir inhibited the loss of primordial follicles. Tumor xenograft studies in mice showed that mangafodipir did not affect anticancer drug antitumor effects.

**Conclusions:**

Oxidative stress might be one of the mechanisms of cisplatin- and paclitaxel-induced the loss of primordial follicles. Mangafodipir can reduce cisplatin- and paclitaxel-induced apoptosis in granulosa cells and primordial follicle activation partially via its SOD activity. At the same time, mangafodipir might have other potential mechanisms to inhibit the activation of primordial follicles. Further, mangafodipir attenuated the ovarian damage caused by cisplatin and paclitaxel without affecting their antitumor activities. Mangafodipir, therefore, though its efficacy might be limited, may be a new option for the preservation of fertility during anticancer treatment.

## Background

Recent progress in anticancer therapy has contributed to improvements in the prognosis of several malignant diseases. However, more female patients of reproductive age have experienced ovarian failure after chemotherapy. It is now considered necessary to take appropriate measures for fertility preservation in both male and female cancer patients who desire future fertility [1]. At present, there are several therapeutic options for preventing female infertility, such as pharmacological protection and the freezing of oocytes or ovaries prior to chemotherapy [2, 3]. Oocyte collection is problematic because ovarian stimulation, which takes several weeks, is typically required, making it unsuitable for women who require urgent treatment. Although significant progress has been made in ovarian cryopreservation technology, the success rate of thawed ovarian transplants remains to be established. Moreover, the autologous transplantation of cryopreserved ovaries might lead to the reintroduction of malignant cells harbored in the ovaries [4]. Technological advancements are therefore required in the handling of ovarian tissue culture in vitro [5] and for the pharmacological protection of ovaries from chemotherapy-induced damage.

The ovarian function of woman is mainly reflected on the amount of primordial follicles in the ovary, while the mechanism of chemotherapy responsible for the loss of primordial follicles remains unclear. It is considered that the chemotherapeutic agents interfere with the various pathways of cell cycle, such as DNA replication and transcription, as well as the formation and function of the spindles and microtubules, which inhibits mitosis of rapidly proliferating cells, leading to the cell damage and apoptosis [6]. Thus, it is concluded that chemotherapeutic drugs may destroy the developing follicles by injuring granulosa cells [7–9]. Another hypothesis is that chemotherapeutic drugs induce apoptosis of follicles, leading to loss of ovarian reserve. Chemotherapeutic agents may act directly on the primordial follicles, leading to damage and apoptosis of primordial follicles [7, 10, 11]. However, it is considered a few cases that anti-tumor drugs directly damage the primordial follicles. Instead, chemotherapeutic drugs indirectly lead to loss of primordial follicles by injuring developing follicles (secondary follicles, and small antral follicles), which is also called the ‘follicular burnout theory’ [12]. Inhibitors of primordial follicular activation are mainly secreted from developing follicles. Therefore, damage and apoptosis of developing follicles caused by chemotherapy decreases such inhibitors, leading to the recruitment of ovarian primordial follicles to developing follicles, resulting in depletion of primordial follicles. It is also reported that the damage and apoptosis were detected in murine oocytes of preantral follicles exposed to cisplatin [13], and secondary follicles exposed to doxorubicin [7, 14]. Meanwhile, in mice ovaries treated with cyclophosphamide, an increased Ki67 immunostaining signals (a proliferation marker) were detected in granulosa cells of early growing follicles, which shows ‘follicular burnout’ theory’ [7, 12]. In addition, it has been reported that chemotherapeutics may damage vessels in the ovarian cortex, thereby affecting the blood circulation of the ovarian cortex and causing destruction of the primordial follicles [9, 15].

It is confirmed that oxidative stress index levels are elevated in primary ovarian insufficiency patients [16]. Moreover, previous literatures indicated that when ovary is exposed to some drugs related to oxidative stress, such as chemotherapeutic drugs, gamma irradiation, or polycyclic aromatic hydrocarbons, loss of primordial follicles is induced. However, the mechanism of reactive oxygen species (ROS) causing primordial follicle depletion is not fully clear yet [17–19]. The mechanisms by which anticancer drugs exert cytotoxicity are diverse, and include the promotion of ROS generation. Low levels of intracellular ROS can stimulate cell growth [20]. However, excessive ROS may result in oxidative stress conditions, leading to interference with the normal function of microtubules and the activation of apoptotic pathways. Oxidative stress is considered to be a significant mechanism involved in chemotherapeutic toxicity, such as that associated with the administration of cisplatin [21], 5-fluorouracil [22], and paclitaxel [23]. Under normal physiological conditions, cells modulate ROS production by balancing its elimination by superoxide dismutase (SOD) [21]. The manganese form of SOD is a homotetrameric protein found in the mitochondrial matrix, and is considered one of the most important enzymes in the cellular defense against oxidative stress [24]. Di Emidio et al. demonstrated that levels of SOD, especially SOD2 are decreased in murine ovaries exposed to chemotherapy and that this effect is prevented by administration of AS101 [25].

Manganese dipyridoxyl diphosphate (mangafodipir; MnDPDP) has been used in magnetic resonance imaging of the liver in humans since 1991 as a diagnostic contrast agent [26]. Previous research has reported that mangafodipir is a chelate of manganese and of the ligand fodipir. Mangafodipir has catalase- and glutathione reductase-like properties as well as SOD activity, allowing it to act at multiple stages of the ROS cascade [27, 28]. The SOD-mimetic activity of mangafodipir was demonstrated using a photometric nitroblue tetrazolium reduction method in 2003 [29], indicating its therapeutic potential.

To date, mangafodipir has been used to treat certain diseases caused by oxidative damage, or to target oxidative damage induced by certain drugs or physical therapy. Furthermore, mangafodipir has potential as a novel preventive treatment for hepatic ischemia-reperfusion injury, as studies in mice showed that it prevented this condition by reducing serum aspartate aminotransferase activity [30]. A case report from 2009 and a recent feasibility study suggest that mangafodipir may prevent or even resolve oxaliplatin-induced peripheral neuropathy in both rats and humans [31, 32], and in vivo studies in mice showed that mangafodipir had a protective effect against paclitaxel-induced leukopenia [28]. These studies support the potential of mangafodipir as an efficacious remedy in pathological conditions caused by oxidative stress. Thus, we hypothesized that mangafodipir might alleviate ovarian damage caused by oxidative stress following treatment with anticancer drugs. The aim of our study was, therefore, to determine whether mangafodipir exerts a protective effect against anticancer drug-induced ovarian damage in vivo and in vitro.

## Methods

### Cell culture

Human non-luteinized granulosa cells (HGrC) that expressed enzymes related to steroidogenesis [33] were maintained in Dulbecco’s modified Eagle’s medium (DMEM; MilliporeSigma, Oakville, Ontario, Canada) containing 10% fetal bovine serum (MilliporeSigma), 100 IU/mL penicillin, 100 g/mL streptomycin, and 25 mg/L amphotericin B. HGrC cells were cultured in 6-well culture plates in humidified air with 5% CO_2_ at 37 °C. In order to enhance the sensitivity of cells to drug stimulation, when the cells reached at least 90% confluence, they were serum starved for 24 h and then cultured in medium containing 1.0 mM hydrogen peroxide (H_2_O_2_), with or without mangafodipir (40, 200, or 1000 μM, MilliporeSigma). The plates were incubated for an additional 24 h before cell morphology was assessed under an optical microscope. Proteins were extracted from the cells for western blotting experiments.

For other experiments, HGrC cells were cultured in 6-well plates and serum starved for 24 h before being cultured in medium containing cisplatin (10 or 60 μM, Nichi-Iko Pharmaceutical Co., Ltd., Toyama, Japan), or paclitaxel (10 or 50 μM, Bristol-Myers Squibb, New York, NY) [34], with or without mangafodipir (200 or 1000 μM). After a further 48 h of incubation, proteins were extracted from the cells for western blotting experiments.

### Animal experiments

Animal studies were approved by the Division of Experimental Animals at Nagoya University Graduate School of Medicine. Female CD-1(ICR) mouse is considered to be suitable for assessment of ovarian function because of its strong reproductive capacity. It has been used in some previous experiments for evaluation of the drug effect on ovarian function [35]. Thus, in order to assess the preventive effect of mangafodipir on cisplatin- and paclitaxel-induced ovarian damage, female CD-1(ICR) mice (Charles River Laboratories Japan, Inc., Kanagawa, Japan) aged 6 weeks (*n* = 4 in each group) were maintained in cages under controlled light conditions (12-h light/12-h dark regimen). After 1 week, mice were injected intraperitoneally with saline or mangafodipir (10 mg/kg body weight). One hour after the mangafodipir injection, mice were injected intraperitoneally with cisplatin (7.5 or 20 mg/kg body weight), paclitaxel (7.5 or 20 mg/kg body weight) [36], or saline. One week later, the mice were killed and their ovaries were removed for histology, immunohistochemistry (IHC), and western blotting analyses.

BALB/c nude mouse has been commonly used for establishment of tumor model in previous studies [37, 38]. Thus, in order to confirm that mangafodipir did not affect the antitumor effects of anticancer drugs, ES-2 cells (human ovarian cancer cell line) were suspended in phosphate buffered saline (PBS) at a density of 1 × 10^7^ cells/mL, mixed with an equal volume of Matrigel (Corning, New York, NY), and injected (200 μL/mouse) subcutaneously into the flanks of 5- to 6-week-old BALB/c (Charles River Laboratories Japan) female nude mice. When palpable tumors were detected, mice were randomly divided into 6 groups (control, mangafodipir without anticancer drugs, 15 mg/kg cisplatin with or without mangafodipir, 7.5 mg/kg paclitaxel with or without mangafodipir; *n* = 4 per group). Mice were then injected intraperitoneally with saline or mangafodipir 1 h prior to the intraperitoneal injection of cisplatin, paclitaxel, or saline (day 0). During the treatment period, tumor growth was monitored every other day (volume = 1/2[length × width^2^]). The rate of increase in tumor volume was calculated for all groups during treatment. On day 5 of treatment, the mice were killed and the rate of tumor volume increase was determined as (tumor volume after treatment − tumor volume before treatment)/tumor volume before treatment × 100%.

### In vitro cell viability assays

HGrC cells (5.5 × 10^3^ cells/well) were seeded in 96-well plates and incubated for 3 h in DMEM containing 10% FBS (200 μL/well) with H_2_O_2_ (0, 0.1, or 1 mM) alone or with mangafodipir (40, 200, or 1000 μM, 4 wells for each group). Thereafter, the media were changed to DMEM with 10% FBS without H_2_O_2_ or mangafodipir, and the cells were incubated in a humidified atmosphere of 5% CO_2_ at 37 °C for 48 h. Separately, HGrC cells (5 × 10^3^ cells/well) were seeded in 96-well plates and incubated for 48 h in DMEM containing 10% FBS with an anticancer agent (10 or 60 μM cisplatin, or 10 or 50 μM paclitaxel) alone or with mangafodipir (200 or 1000 μM) in a humidified atmosphere of 5% CO_2_ at 37 °C. The culture media of both groups of HGrC cells were then replaced with 100 μL DMEM per well, and 20 μL of methyltrichlorosilane (MTS) solution (CellTiter 96® AQueous One Solution Cell Proliferation Assay, Promega, Madison, WI) was added to each well. The plates were incubated for 2 h, and the absorbance at 490 nm was measured using a microplate reader (BioTek, Winooski, VT).

### Follicle counting and IHC

Ovaries removed from treated female CD-1(ICR) mice were fixed in 10% formalin and embedded in paraffin. Serial sections (6 μm thick) were collected, and every twentieth section was selected for staining with hematoxylin and eosin for follicle counting. Approximately 10–16 sections were needed from each mouse ovary for the follicle count. Follicles were classified into four stages. A primordial follicle was defined as the presence of flattened granulosa cells (GCs) only. A primary follicle was defined as the presence of cuboidal GCs with or without flattened GCs. Secondary follicles were defined as having two or more layers of cuboidal granulosa cells with no visible antrum. Follicles were considered to be antral follicles when the follicle possessed an antral space containing follicular fluid. [35]. Follicle health was assessed using standard morphological criteria: follicles were defined as unhealthy if they had (i) an oocyte with eosinophilic, shrunken or non-homogeneous cytoplasm, or condensed nuclear chromatin; (ii) GCs with condensed chromatin or irregular shapes; or (iii) an unhealthy oocyte and GCs [39].

Immunohistochemical analysis of cleaved caspase-3 was carried out to measure the amount of apoptosis. Measurement of the end products of lipid peroxidation is a widely accepted assay for oxidative stress. 4-hydroxynonenal (4-HNE), an aldehydic secondary product of lipid peroxidation, is an established marker of oxidative stress [40] and was therefore used to evaluate the generation of ROS in mouse ovarian tissue. We selected the ovarian section with the middle number of all the serial sections. There were four ovaries in each group that were used for follicle counting, and we put four ovaries of each group in the same paraffin embedded block. We counted the number of all follicles in each stage for every ovary present in this section. The count and score of the four ovaries in this section were then averaged to provide the result. In brief, after standard deparaffinization and rehydration, selected sections were immersed in citrate buffer (pH 6.0), and incubated (microwave, 95 °C, 20 min) for antigen retrieval. Then the selected sections were incubated in 0.3% H_2_O_2_ for 20 min and then in blocking solution (10% normal goat serum) for 1 h at room temperature. We confirmed that 0.3% H_2_O_2_ and 3% H_2_O_2_ for blocking endogenous peroxidase activity bring a similar result in the preliminary experiments. Next, sections were reacted with rabbit anti-cleaved caspase-3 antibody (1:100; Cell Signaling Technology Japan, K.K., Tokyo, Japan), rabbit anti-4-HNE antibody (1:1500; ab46545; Abcam, Tokyo, Japan) and rabbit anti-Ki67 antibody (1:500; ab92742; Abcam, Cambridge, UK) overnight at 4 °C. After washing in PBS, slides were incubated for 10 min at room temperature with a biotinylated secondary goat anti-rabbit antibody (Nichirei Bioscience, Tokyo, Japan), washed in PBS, incubated with avidin-biotin-peroxidase solution (Nichirei Bioscience) for 5 min, visualized by staining with 3,3′-diaminobenzidine (Agilent, Santa Clara, CA), and counterstained with hematoxylin.

For the semi-quantification of cleaved caspase-3, the immunoreactive density was scored. The scoring standard for cleaved caspase-3 staining was as follows: percentage of positive GCs in a follicle, 0, 0%; 1, 0–10%; 2, 10–50%; and 3, 50–100%. The scoring standard for 4-HNE staining was as follows: oocyte staining density, weak = 1, moderate = 2, strong = 3; and percentage of positive GCs in a follicle, 0, 0%; 1, 0–50%; and 2, 50–100%. Finally, we calculated the total score for all follicles in all ovaries from each group. Primordial follicles with at least one Ki-67-positive granulosa cell or oocyte were defined as Ki-67-positive follicles, and the percentage of Ki-67-positive primordial follicles in all primordial follicles was calculated.

### Western blot analysis

Equal amounts of proteins were mixed with sample buffer (4% sodium dodecyl sulfate (SDS), 10% beta-mercaptoethanol, and 20% glycerol in 0.125 M Tris, pH 6.8) containing bromophenol blue and boiled for 5 min. The samples were loaded and separated via 10% SDS-polyacrylamide gel electrophoresis (PAGE). Proteins separated via SDS-PAGE were transferred to polyvinylidene difluoride membranes (Immobilon-P transfer membrane) and blocked for 1 h with blocking buffer (5% non-fat milk in tris-buffered saline containing 0.5% Tween-20 (TBST) for 1 h. Next, the membranes were incubated overnight at 4 °C with primary antibodies for anti-cleaved caspase-3 (1:500; Cell Signaling Technology), anti-4-HNE (1:500; ab46545, Abcam), and β-actin (1:10000; no. 017–24573, Wako Pure Chemical Industries Ltd., Tokyo, Japan). Membranes were washed three times with Tween/PBS for 15 min, and then incubated with anti-rabbit immunoglobulin G (IgG) (1:1000; Cell Signaling Technology) for 1 h. Finally, membranes were washed with Tween/PBS and treated with ECL-western blot detecting reagent (Amersham Biosciences Corp., Piscataway, NJ) for visualization. β-actin was used as an internal control.

### Statistical analysis

Statistically significant differences between two groups were determined using Student’s *t*-test or the Mann–Whitney *U*-test. The latter was applied when the variables were not normally distributed. One-way ANOVA with Bonferroni’s pairwise multiple comparison test was used for comparisons among more than three groups. Values are expressed as the mean ± SEM or SD. A *p*-value of less than 0.05 was considered statistically significant. All analyses were performed using SigmaPlot13 software (Systat Software Inc., San Jose, CA).

## Results

### Mangafodipir inhibits H_2_O_2_- and anticancer drug-induced apoptosis in GCs

**Fig. 1:**
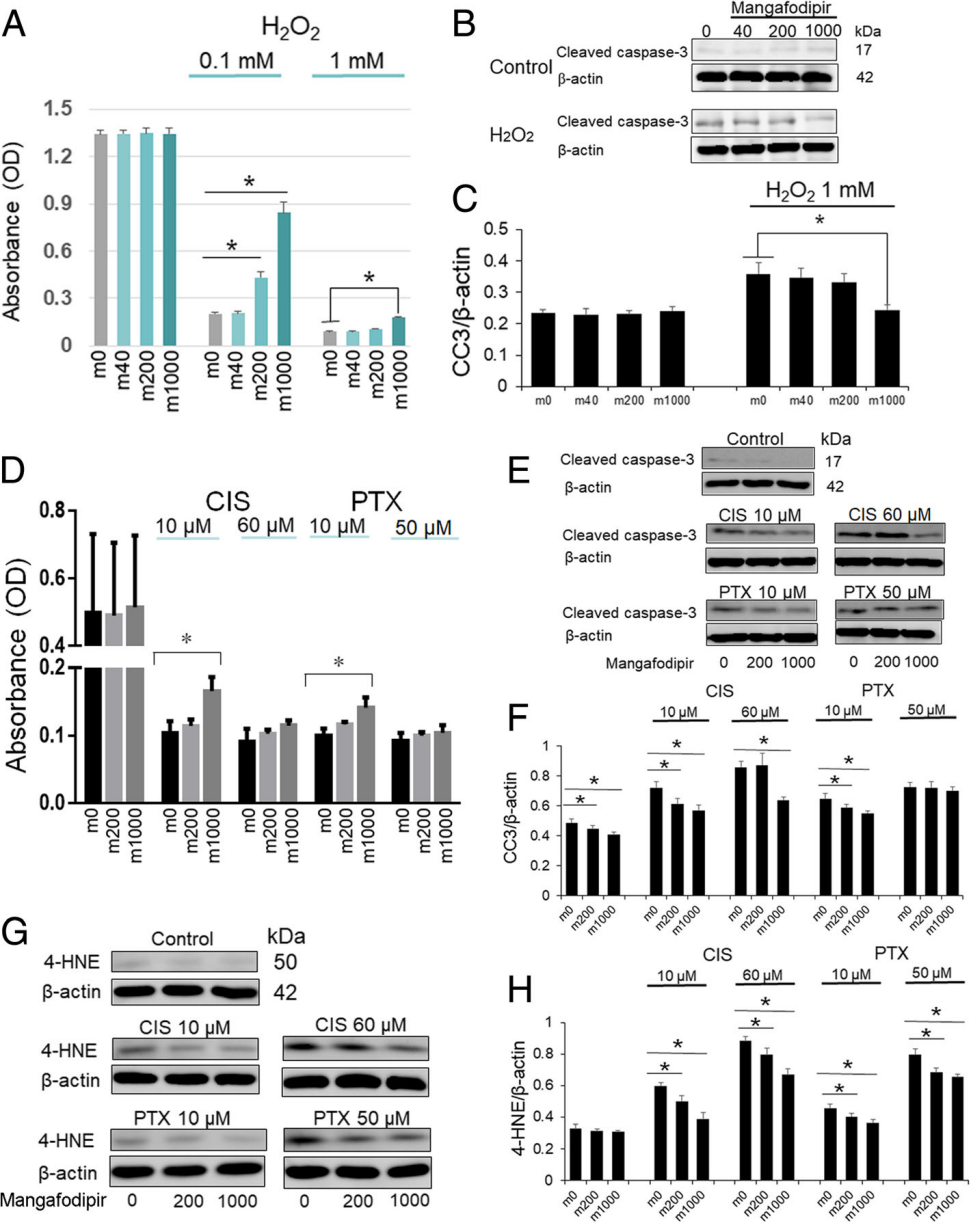
Mangafodipir inhibits apoptosis in granulosa cells induced by H_2_O_2_ and anticancer drugs. **a** A MTS assay was performed after treatment with H_2_O_2_ (0, 0.1, or 1 mM) without (m0) or with the administration of mangafodipir (40 μM: m40; 200 μM: m200; 1000 μM: m1000). Mean ± SEM, *, *p* < 0.01 vs. each control (m0). **b** Apoptosis was assessed via immunoblotting using anti-cleaved caspase-3 antibody. Mangafodipir did not increase cleaved caspase-3 levels (upper panels). Cleaved caspase-3 induced by 1 mM H_2_O_2_ was suppressed by the simultaneous addition of 1000 μM/L mangafodipir (lower panels). **c** The relative intensity of cleaved caspase-3 (CC3) to β-actin was measured to assess the results of the immunoblot in the H_2_O_2_ in vitro experiments. Mean ± SEM, *, *p* < 0.01 vs. each control (m0). **d** A MTS assay was performed after 48-h treatment with 10 or 60 μM cisplatin (CIS) or 10 or 50 μM paclitaxel (PTX) with mangafodipir (200 μM: m200; 1000 μM: m1000) or without mangafodipir (m0). Mean ± SEM, *, *p* < 0.05 vs. each control (m0). **e** Apoptosis was assessed via immunoblotting using anti-cleaved caspase-3 antibody. Cleaved caspase-3 was induced with 10 or 60 μM cisplatin (CIS) and 10 μM paclitaxel (PTX) and was suppressed by the simultaneous addition of 1000 μM mangafodipir. **f** The relative intensity of cleaved caspase-3 (CC3) to β-actin was measured to assess the results of the immunoblot in the anticancer drug-based in vitro experiments. Mean ± SEM, *, *p* < 0.05 vs. each control (m0). **g** ROS production was assessed by immunoblotting using anti-4HNE antibody. 4-HNE induced by 10 or 60 μM cisplatin (CIS) or 10 or 50 μM paclitaxel (PTX) was suppressed by the simultaneous addition of 200 μM and 1000 μM mangafodipir. **h** The relative intensity of 4-HNE to β-actin was measured to assess the results of immunoblotting in anticancer drugs in vitro experiments. Mean ± SEM, *, *p* < 0.05 vs. each control (m0)

HGrC cells were treated with mangafodipir at concentrations of 40, 200, and 1000 μM with or without H_2_O_2_. MTS assay results showed that mangafodipir did not affect the proliferation and viability of HGrC cells that were not treated with H_2_O_2_. However, mangafodipir (200 or 1000 μM) did attenuate the suppression of HGrC cell viability caused by 0.1 mM H_2_O_2_, and mangafodipir (1000 μM) attenuated the inhibition of HGrC cell viability caused by 1 mM H_2_O_2_ (Fig. 1a). In addition, the results of immunoblotting indicated that 1 mM H_2_O_2_ treatment significantly increased the levels of cleaved caspase-3, and the simultaneous addition of 1000 μM mangafodipir significantly suppressed this induced apoptosis. However, no effects due to mangafodipir (any concentration) were observed in non-H_2_O_2_ treated control cells, nor did 40 or 200 μM mangafodipir cause any effects in 1 mM H_2_O_2_-treated cells (Fig. 1b and c).

Next, we investigated the effect of mangafodipir on anticancer drug-induced cytotoxicity in HGrC cells. Cell survival was assessed after 48 h-treatments with 10 or 60 μM cisplatin or 10 or 50 μM paclitaxel in the presence (200 or 1000 μM) or absence of mangafodipir. The suppression in viability of HGrC cells caused by low-dose cisplatin and low-dose paclitaxel was significantly attenuated by the simultaneous addition of mangafodipir at a concentration of 1000 μM (Fig. 1d). The cleavage of caspase-3 induced by 10 μM cisplatin or paclitaxel was suppressed by the simultaneous addition of mangafodipir at concentrations of 200 and 1000 μM, and 1000 μM mangafodipir attenuated the apoptosis caused by 60 μM cisplatin. However, mangafodipir did not significantly suppress cleaved caspase-3 levels in HGrC cells treated with 50 μM paclitaxel (Fig. 1e and f). In contrast, 4-HNE levels induced by cisplatin (10, 60 μM) or paclitaxel (10, 50 μM) were significantly suppressed by the simultaneous addition of mangafodipir at concentrations of 200 and 1000 μM (Fig. 1g and h).

### Effect of mangafodipir on the number of follicles at each developmental stage and on the rate of healthy follicles in anticancer drug-treated mice ovaries

**Fig. 2:**
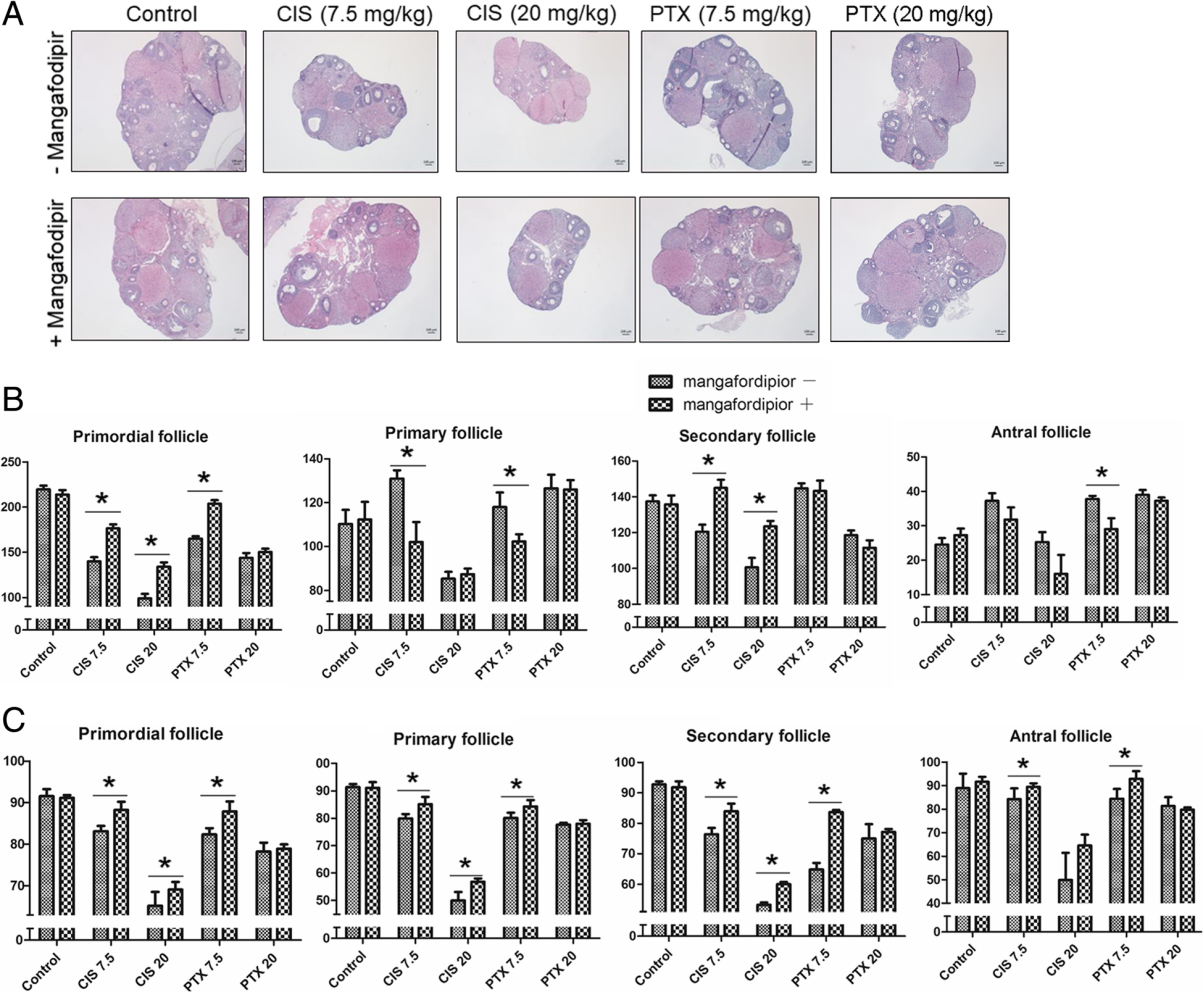
Effects of mangafodipir on the number of follicles at each development stage and on the rate of healthy follicles after anticancer treatment. **a** Representative images of ovaries on day 7 after the administration of 7.5 or 20 mg/kg cisplatin (CIS), or 7.5 or 20 mg/kg paclitaxel (PTX), with or without mangafodipir (+/− Mangafodipir, respectively). Bar, 100 μm. **b** The total number of follicles at each developing stage (primordial, primary, secondary, antral) after the administration of 7.5 (CIS7.5) or 20 (CIS20) mg/kg cisplatin, 7.5 (PTX7.5) or 20 (PTX20) mg/kg paclitaxel, with or without mangafodipir (+/− Mangafodipir, respectively). Mean ± SD, *, *p* < 0.01. **c** The rates of healthy follicles after the administration of cisplatin and paclitaxel, with or without mangafodipir. Mean ± SD, *, *p* < 0.05

Selected ovarian sections of mangafodipir-treated mice with or without anticancer drugs were stained and examined under a light microscope (Fig. 2a), and the number of ovarian follicles was counted for each developmental stage. All anticancer drugs caused a decrease in the number of primordial follicles, with the high-dose cisplatin group showing the most significant loss of primordial follicles (Fig. 2b). The simultaneous administration of mangafodipir attenuated the loss of primordial follicles caused by 7.5 or 20 mg/kg cisplatin and 7.5 mg/kg paclitaxel, while there was no obvious change on the loss of primordial follicles in the high-dose paclitaxel group. With the exception of the high-dose cisplatin group, the anticancer agents increased the number of primary follicles, and the addition of mangafodipir prevented a change in the number of primary follicles induced by 7.5 mg/kg cisplatin or 7.5 mg/kg paclitaxel. Cisplatin and 20 mg/kg paclitaxel also caused a reduction in the number of secondary follicles. The addition of mangafodipir attenuated the reduction of secondary follicles in the cisplatin groups, but not in the paclitaxel groups. There were increases in the number of antral follicles in the 7.5 mg/kg cisplatin and paclitaxel groups, and addition of mangafodipir attenuated the increase of antral follicles in the 7.5 mg/kg paclitaxel group (Fig. 2b). We next analyzed the number of healthy follicles at each stage by using a morphological evaluation. For primordial, primary or secondary follicles, mangafodipir alleviated the follicular damage caused by 7.5 or 20 mg/kg cisplatin or 7.5 mg/kg paclitaxel. Mangafodipir also attenuated the damage of antral follicles in the 7.5 mg/kg cisplatin group and 7.5 mg/kg paclitaxel group, while there was no significant differences in the high-dose paclitaxel group (Fig. 2c).

### Mangafodipir protects follicles from anticancer drug-induced apoptosis and oxidative stress, and prevents the proliferation of the primordial follicles

**Fig. 3:**
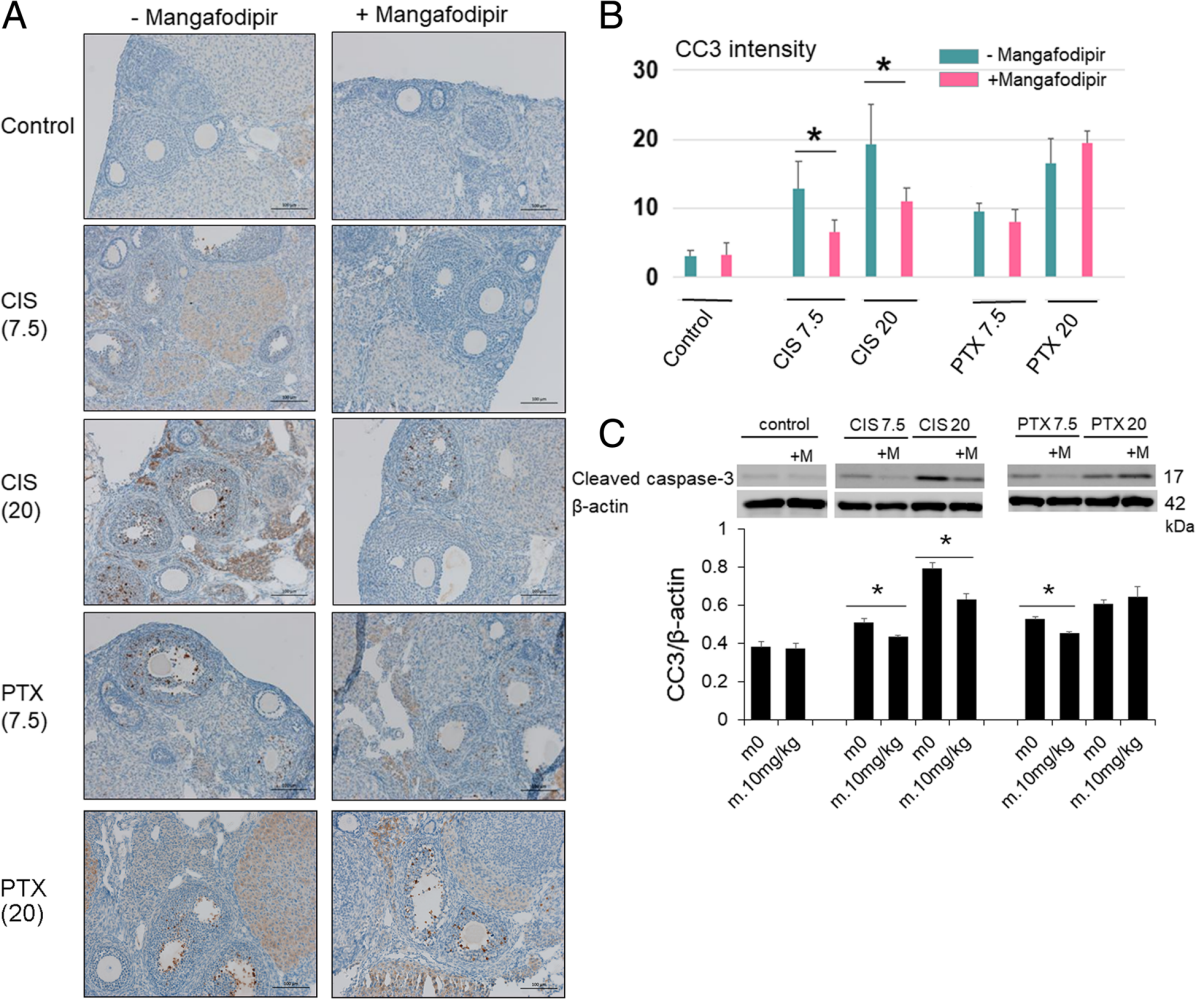
Mangafodipir rescues follicles from anticancer drug-induced apoptosis. **a** Representative images of immunohistochemistry for cleaved caspase-3 in ovaries treated with 7.5 (CIS7.5) or 20 (CIS20) mg/kg cisplatin, or 7.5 (PTX7.5) or 20 (PTX20) mg/kg paclitaxel, with or without mangafodipir (+/− Mangafodipir, respectively). Bar, 100 μm. **b** Cleaved caspase-3 (CC3) immunoreactive intensity scores. Mean ± SD, *, *p* < 0.05. **c** Mangafodipir (m.10 mg/kg) significantly reduced cleaved caspase-3 levels induced by 7.5 (CIS7.5) or 20 (CIS20) mg/kg cisplatin, 7.5 (PTX7.5) mg/kg paclitaxel, but not by 20 (PTX20) mg/kg paclitaxel. The relative intensities of cleaved caspase-3 (CC3) signals were normalized to those of β-actin to assess the results of immunoblot in mice ovaries. Mean ± SEM, *, *p* < 0.05 vs. each control (m0)

Cleaved caspase-3-positive follicles were detected in the ovaries of mice treated with anticancer drugs (Fig. 3a). A semi-quantitative analysis of the cleaved caspase-3 signal detected by IHC showed a significant decrease when mice were treated with mangafodipir and 7.5 or 20 mg/kg cisplatin. We also found a decrease in the 7.5 mg/kg paclitaxel group without significant change (*p* = 0.051). No changes in caspase-3 signals were found when mangafodipir was given with 20 mg/kg paclitaxel (Fig. 3b). Cleaved caspase-3 was detected in the western blots of mouse ovary tissue extracts. The addition of mangafodipir significantly reduced the increase in cleaved caspase-3 levels induced by 7.5 or 20 mg/kg cisplatin or 7.5 mg/kg paclitaxel, but did not affect the increase in cleaved caspase-3 caused by 20 mg/kg paclitaxel (Fig. 3c). Due to the limitation of loading wells, the control samples and experimental samples were not always applied in the same gels. However, we obtained the similar results regardless of whether we applied the samples in the same gels or not. Similarly, cleaved caspase-3 signals in ovarian sections were assessed using IHC and were found to be mainly localized in the GCs of secondary and small antral follicles. However, cleaved caspase-3 was virtually undetectable in the GCs of primordial, primary, and large pre-ovulatory follicles. A negligible signal for cleaved caspase-3 was also detected in the absence of anticancer treatment, regardless of the presence or absence of mangafodipir.

**Fig. 4:**
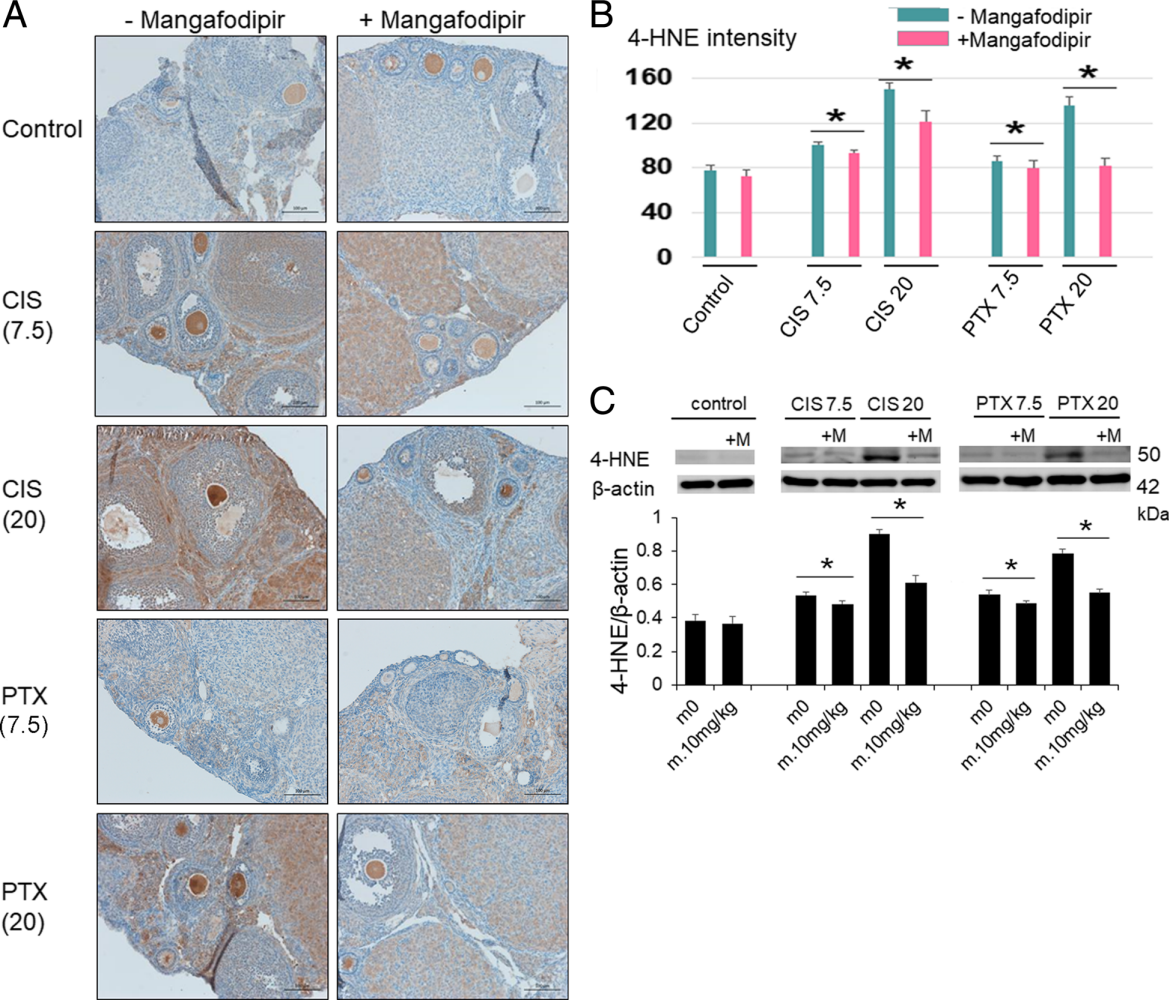
Mangafodipir rescues follicles from anticancer drug-induced oxidative stress. **a** Representative images of immunohistochemistry for 4-HNE in ovaries treated with 7.5 (CIS7.5) or 20 (CIS20) mg/kg cisplatin, or 7.5 (PTX7.5) or 20 (PTX20) mg/kg paclitaxel, with or without mangafodipir (+/− Mangafodipir, respectively). Bar, 100 μm. **b** 4-HNE immunoreactive intensity scores. Mean ± SD, *, *p* < 0.05. **c** Mangafodipir (m.10 mg/kg) reduced 4-HNE induced by 7.5 (CIS7.5), 20 (CIS20) mg/kg cisplatin, 7.5 (PTX7.5) or 20 (PTX20) mg/kg paclitaxel. The relative intensities of 4-HNE signals were normalized to those of β-actin to assess the results of the immunoblot in mice ovaries. Mean ± SEM, *, *p* < 0.05 vs. each control (m0)

For the evaluation of ROS, the IHC for 4-HNE showed that 4-HNE levels were significantly increased in the ovaries of mice treated with cisplatin and paclitaxel. Therefore, we can infer that cisplatin and paclitaxel are closely associated with the generation of ROS. The addition of mangafodipir reduced the 4-HNE signal in the ovaries of mice treated with all anticancer drugs, especially those treated with high-dose cisplatin and high-dose paclitaxel. This result suggests that mangafodipir exerts SOD activity.. Further, oocytes presented stronger expressions of 4-HNE than those in granulosa cells (Fig. 4a). We scored the follicles on the basis of oocyte density and the percentage of immunopositive granulosa cells. The results showed that the scores for immunoreactive density were significantly increased in the cisplatin groups as well as in the paclitaxel groups; the addition of mangafodipir decreased these scores (Fig. 4b). Moreover, Western blotting showed similar results to those obtained by IHC (Fig. 4c).

**Fig. 5:**
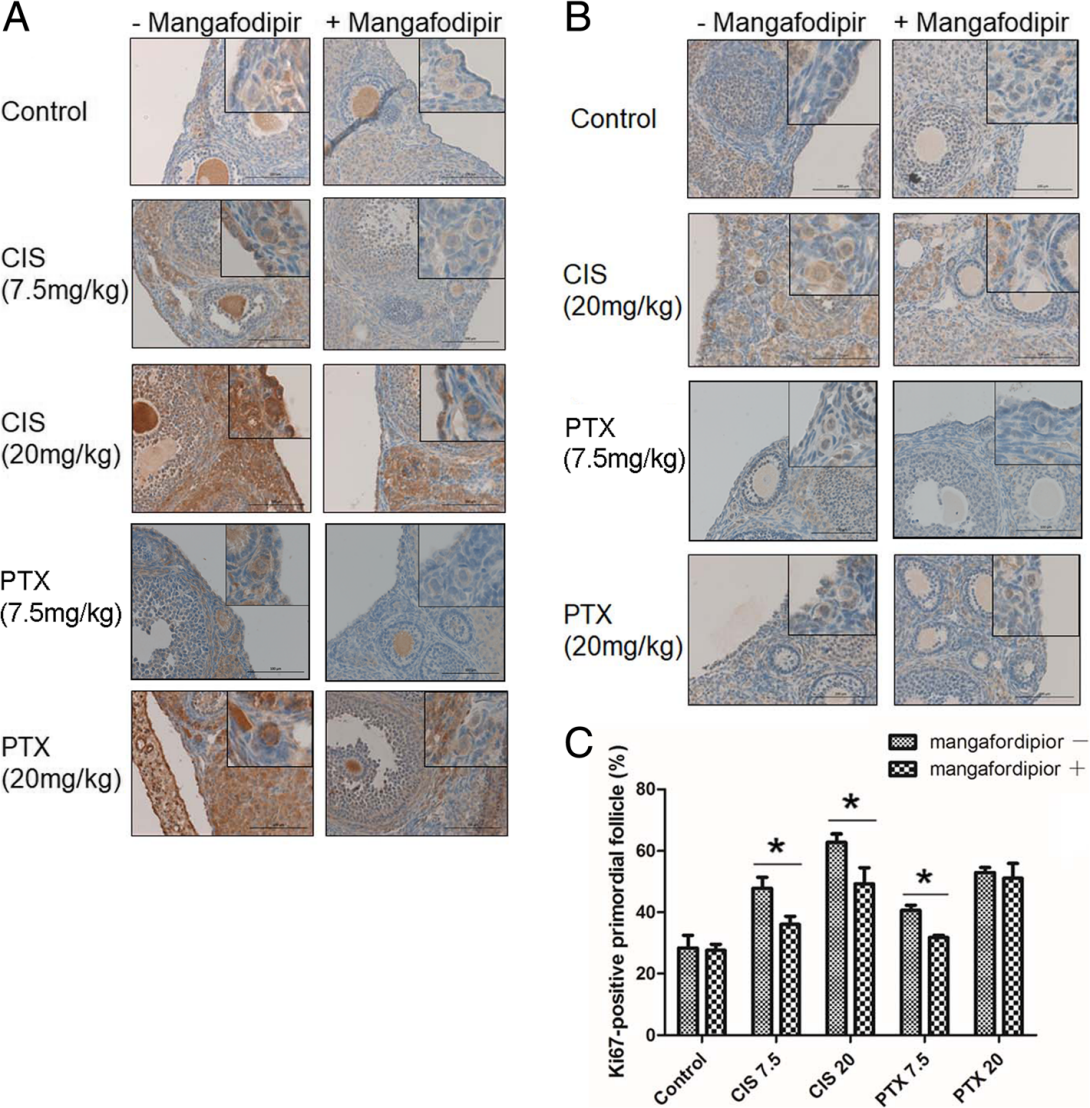
Expressions of 4-HNE and Ki67 in primordial follicles. **a** Representative images of immunohistochemistry for 4-HNE in primordial follicles of murine ovaries treated with 7.5 (CIS7.5) or 20 (CIS20) mg/kg cisplatin, 7.5 (PTV7.5) or 20 (PTX20) mg/kg paclitaxel, with or without mangafodipir (+/− Mangafodipir, respectively). Bar, 100 μm. **b** Representative images of immunohistochemistry for Ki67 in primordial follicles of murine ovaries treated with 20 (CIS20) mg/kg cisplatin, or 7.5 (PTX7.5) or 20 (PTX20) mg/kg paclitaxel, with or without mangafodipir (+/− Mangafodipir, respectively). Bar, 100 μm. **c** The percentage of Ki67-positive primordial follicles. Mean ± SEM, *, *p* < 0.05

To explore the relationship between antioxidant activity of mangafodipir and proliferation of primordial follicles, we observed the expressions of 4-HNE and Ki67 in primordial follicles. The results of IHC indicated that 4-HNE was weakly expressed in the primordial follicles in the control group, and its expression was not changed obviously after mangafodipir was added. In 7.5, 20 mg/kg cisplatin and 20 mg/kg paclitaxel groups, significant expressions of 4-HNE were observed in granulosa cells and oocytes of primordial follicles. At the same time, the addition of mangafodipir significantly reduced the expression of 4-HNE on the primordial follicles in cisplatin and paclitaxel groups (Fig. 5a). On the other hand, there were no obvious expressions of Ki67 detected in primordial follicles in the absence of anticancer drugs. 7.5 and 20 mg/kg cisplatin significantly enhanced the expression of Ki67 in granulosa cells and oocytes of primordial follicles, and these strong Ki67 signals were significantly weakened after mangafodipir treatment. Although 7.5 mg/kg paclitaxel also enhanced the expression of Ki67 in primordial follicles, it was weaker than that of 7.5 mg/kg cisplatin. The addition of mangafodipir did not obviously change the expression of Ki67 in the primordial follicles in 20 mg/kg paclitaxel group (Fig. 5b and c).

### Mangafodipir does not impair the antitumor effects of anticancer drugs

**Fig. 6:**
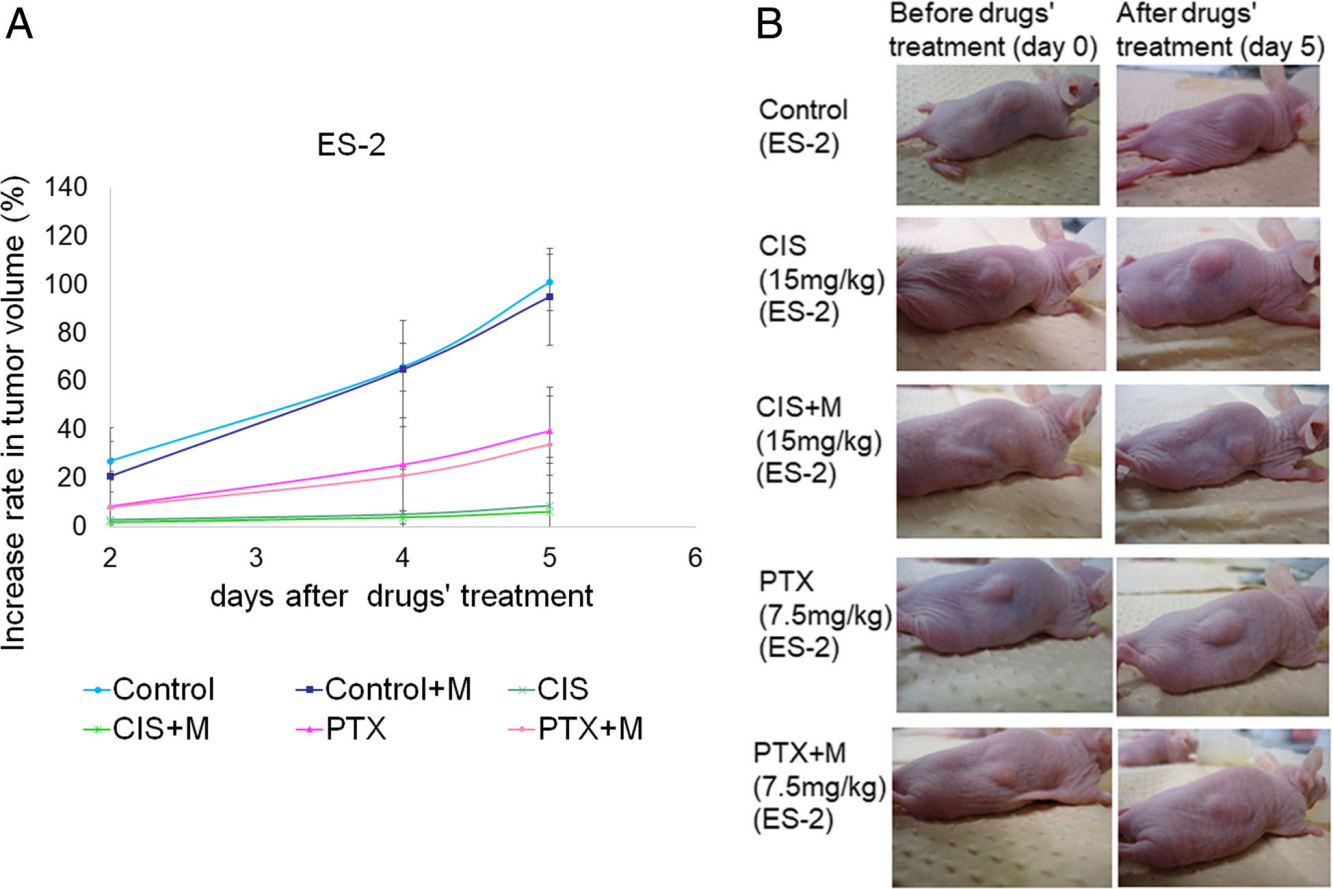
Mangafodipir does not impair the antitumor effects of anticancer drugs. Nude mice were injected with 15 mg/kg cisplatin or 7.5 mg/kg paclitaxel with or without mangafodipir (day 0, PTX, CIS, PTX + M, and CIS + M) and sacrificed 5 days after drug injection (day 5). **a** Increase rate of tumor sizes of ES-2 tumors in nude mice after drug’s treatment. Mean ± SD. **b** Representative pictures showing tumors in nude mice prior to treatment (day 0) and after treatment (day 5)

In nude mice treated with 7.5 mg/kg paclitaxel, the growth rates of ES-2 tumors were significantly lower than those of the control group. Similarly, treatment with 15 mg/kg cisplatin significantly inhibited tumor growth. The addition of mangafodipir did not affect the antitumor effect of cisplatin or paclitaxel (Fig. 6a and b).

## Discussion

Premature ovarian failure (POF) is mainly manifested by a depletion in the number of primordial follicles in women’s ovaries. According to the latest reports, gene mutations that cause breast cancer susceptibility and the genetic defect fragile X syndrome might be associated with ovarian aging and POF. Other causes of POF may include radiotherapy, chemotherapy, and surgical treatment in the ovaries [41]. Chemotherapy can improve survival among female cancer patients, although it may damage ovarian function to some extent and affect fertility. Oocyte cryopreservation and ovarian tissue cryopreservation have become widespread strategies for female fertility preservation [42, 43]. However, oocyte cryopreservation requires assisted reproduction, such as in vitro fertilization, and ovarian tissue cryopreservation requires surgery for the collection of ovarian tissues as well as for autologous transplantation. Therefore, the development of simpler procedures, for use alone or in combination with fertility cryopreservation, is of clinical importance. The pharmacological protection of the ovary during chemotherapy has been investigated as one such procedure. However, few candidate drugs have been identified, with the exception of gonadotropin-releasing hormone agonists, the efficacy of which remains controversial as studies are inconsistent [44, 45]. In addition, it has been reported recently that Müllerian inhibiting substance (or anti-Müllerian hormone/AMH) can prevent chemotherapy-induced primary ovarian insufficiency by blocking the activation of primordial follicles in C57BL/6 N mice [46]. AS101 [ammonium trichloro (dioxoethylene-o, o′) tellurate] is another drug candidate. It is a nontoxic immunomodulatory compound that has been reported to suppress up-regulation of the PI3K/PTEN/Akt follicle activation pathway caused by cyclophosphamide in BALB/c mice [12] and CD1 mice [25].

According to previous reports, the production of excessive ROS contributes to the pharmacological effects of some chemotherapeutic drugs, including cisplatin [21] and paclitaxel [23]. Our results showed that 4-HNE, a marker of ROS production, was significantly increased in cisplatin- and paclitaxel-treated mice ovaries, especially in the high-dose cisplatin group. Excessive ROS not only damages cancer cells, but also induces apoptosis through both the extrinsic and intrinsic apoptotic pathways in normal cells [47]. The mitochondrion is the primary target of cisplatin-induced oxidative stress, followed by the loss of the mitochondrial protein sulfhydryl group, inhibition of calcium uptake, and reduction of membrane potential in the mitochondria, leading to a release of cytochrome c into the cytosol resulting in subsequent activation of the caspase pathway and thus apoptosis [48]. In addition, it is considered that oxidative stress has a strong connection with endoplasmic reticulum stress, and cisplatin also causes endoplasmic reticulum stress, with subsequent activation of caspase 12 and apoptosis [49, 50]. Moreover, previous literature has reported that oxidative stress can aggravate DNA damage and affect the energy metabolism of cells, which can lead to cell damage. Thus, we think oxidative damage might be one of the causes of harm to follicles [47]. In the current study, IHC results for 4-HNE showed that the expression of 4-HNE can be detected in oocytes and granulosa cells of follicles. It seems that oocytes had a stronger signal than granulosa cells, likely because of the large number of mitochondria in the cytoplasm of oocytes. IHC results for cleaved caspase-3 also confirmed the apoptosis of granulosa cells in follicles. Thus, we inferred that free radicals might cause damage and apoptosis in follicles by injuring granulosa cells, although the specific mechanism still needs to be determined. In addition, IHC results for the expressions of 4-HNE and Ki67 in primordial follicles show that anticancer drug increases the oxidative stress in the primordial follicles, and at the same time the primordial follicles also show corresponding proliferation. Therefore, we speculate whether oxidative stress can directly stimulate the activation of the primordial follicles, which needs further research to verify.

Our results also showed that caspase-3 cleavage was increased following treatment with cisplatin and paclitaxel. Cleaved caspase-3 was mainly localized in the granulosa cells of developing follicles, but rarely observed in primordial or primary follicles. This is consistent with the report of Fenwick et al. [51]. Moreover, the results of follicular counting showed that the number of primordial follicles decreased after treatment with these two anticancer drugs. These results suggest that these anticancer drugs induce the apoptosis of granulosa cells in follicles. In addition, ROS and other toxicities produced by the anticancer drugs are more likely to damage the developing follicles than primordial follicles. Since the inhibitory factors for primordial follicle activation (such as AMH and inhibin B) are mainly secreted from the granulosa cells of developing follicles [52, 53], we inferred that the damage and apoptosis seen in developing follicles might inhibit the secretion of these inhibitory factors, resulting in a loss of primordial follicles (Fig. 6).

**Fig. 7:**
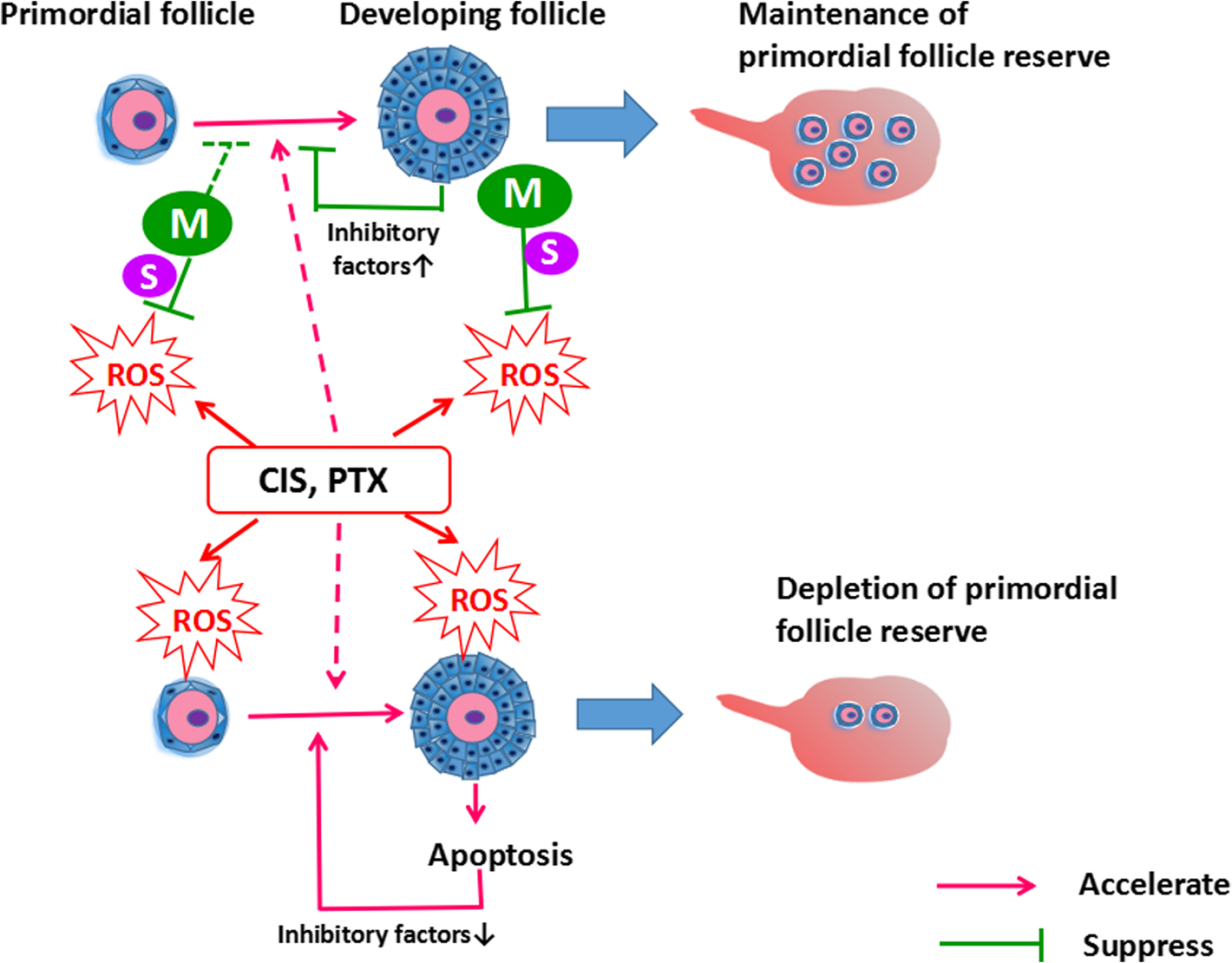
Mechanism of mangafodipir on maintaining the primordial follicle reserve. Excessive ROS produced by cisplatin (CIS) and paclitaxel (PTX) led to apoptosis of developing follicles. Apoptosis in developing follicles reduced the amount of inhibitory factors secreted by granulosa cells that activate primordial follicles (such as AMH and inhibin B), and accelerated the activation of primordial follicles and the loss of ovarian reserve. Mangafodipir (M), that possesses SOD2 activity (S), attenuated ROS-induced damage to follicles. Thus, inhibitory factors secreted by developing follicles can inhibit the activation of primordial follicles and maintain normal ovarian reserve. Cisplatin and paclitaxel might have other mechanisms to activate primordial follicles (pink dotted line). And mangafodipir might have other mechanisms that can directly inhibit the activation of primordial follicles (green dotted line)

Our findings demonstrated that mangafodipir attenuates the generation of ROS in ovaries and cisplatin- and paclitaxel-induced apoptosis in granulosa cells. Therefore, we propose that mangafodipir prevents ovarian damage induced by cisplatin and paclitaxel in vivo partially via its SOD activity. We have recently reported that H_2_O_2_-induced granulosa cell apoptosis can be rescued with sphingosine-1-phosphate [54]. Similarly, our in vitro experiments showed that mangafodipir can inhibit H_2_O_2_-induced granulosa cell apoptosis. Taken together, our results indicated that upregulation of ROS in the ovary led to apoptosis in granulosa cells, which is followed by the loss of follicles. Mangafodipir can inhibit apoptosis in granulosa cells, resulting in a reduction in the loss of follicles. Moreover, suppression on damages and apoptosis of developing follicles by mangafodipir, leading to an enough secretion of inhibitory factors to inhibit the activation of primordial follicles and maintaining normal ovarian reserve (Fig.7). The results of our experiment on evaluation of follicular health also confirm that mangafodipir attenuates damage caused by anticancer drugs in secondary follicles (developing follicles). On the other hand, our results show that mangafodipir can significantly reduce the strong expressions of 4HNE in the primordial follicles induced by cisplatin and paclitaxel. And mangafodipir can also significantly weaken the strong signal of Ki67 in the primordial follicles caused by 20 mg/kg cisplatin. Therefore, it suggests that the antioxidant activity of mangafodipir is partially related to its inhibitory effects on the activation of primordial follicles caused by anticancer drugs. Therefore, the administration of mangafodipir during chemotherapy with cisplatin and/or paclitaxel may represent a promising strategy for the pharmacological protection of the ovary.

However, our results showed that the antioxidant effect of mangafodipir in the ovaries of mice treated with high-dose of cisplatin or paclitaxel was more significant than that in low doses of anti-cancer drugs groups. However, follicular counts and Ki67 immunohistochemistry showed that in the low-dose paclitaxel group, the inhibitory effect of mangafodipir on primordial follicular activation was more significant than that in the high-dose group. At the same time, the results of IHC for 4-HNE and Ki67 suggested that in the high-dose paclitaxel group, although mangafodipir significantly decreased the expression of 4-HNE in primordial follicles, it did not significantly reduce the expression of Ki67 in primordial follicles. Follicle counts also shows a consistent result. That is to say, in the high-dose paclitaxel group, the antioxidant effect of mangafodipir failed to effectively inhibit the activation of primordial follicles. Therefore, we infer that about the mechanisms of paclitaxel on the activation of the primordial follicles and the ovarian damage, it has other more important mechanisms to activate the primordial follicles than oxidative damage. Although the antioxidant activity of mangafodipir can attenuate the activation of the primordial follicles caused by oxidative damage in the high-dose paclitaxel group, it is not sufficient to antagonize other actions of paclitaxel on the activation of the primordial follicles. In addition, although the antioxidant effect of mangafodipir in the low-dose paclitaxel group is weaker than that in the high-dose group, mangafodipir has a significant inhibitory effect on the activation of the primordial follicles caused by low-dose paclitaxel. Regarding the reason, we infer that not only oxidative stress, but also other actions of paclitaxel on the activation of the primordial follicles are significantly lower in the low dose than group than those in the high dose group. In addition, we speculate that, mangafodipir might have other mechanisms to directly inhibit the primordial follicle activation, than SOD activity [12]. Further research is needed to clarify all the mechanisms used.

Some previous studies have demonstrated that mangafodipir can enhance the therapeutic index of anticancer agents by protecting normal cells and increasing the antitumor activity of these agents [55, 56]. Our results showed that mangafodipir did not affect the antitumor effects of cisplatin or paclitaxel on ES-2 tumors in a nude mice model. The mechanism by which mangafodipir protects normal cells but damages cancer cells remains unclear. Some researchers have inferred that the cytotoxic activity of mangafodipir is an inherent property of its chelator moiety alone, and not of the intact Mn^2+^ complex. This might explain why cancer cells are more likely to take up the dissociated chelator, while the intact Mn^2+^ complex is preferably taken up by normal cells [57, 58]. However, this observation requires further clarification prior to commencing clinical studies of mangafodipir.

Regarding the dosage of anticancer drugs, we partially referred to the doses used in the previous literatures [34, 36]. However, the dosage were actually decided in our experiment based on the effects observed in the experiments We chose the dose and treatment time of mangafodipir based on results from the relevant literature. This dosage (10 mg/kg) of mangafodipir is the commonly accepted dose used in the past mangafodipir experiments, and is the dosage of mangafodipir used in clinical MRIs [58]. As a drug that protects the function of the ovary, the extent to which mangafodipir can be effective depends not only on the properties of mangafodipir itself, but also on its interactions with various antitumor drugs. Based on our experimental results, we cannot say if mangafodipir is effective when given concurrently with many kinds of anticancer drugs, although we have shown that it can prevent ovarian injury and the loss of the primordial follicle caused by cisplatin and paclitaxel to a certain extent.

Another limitation of our study is the low number of mice used for in vivo experiments. Increasing the number of mice for in vivo experiments might allow for more accurate results. However, some previous reports similarly allocated 3–5 mice in each group [59, 60]. In addition, 10–16 sections from each mouse ovary were selected and counted after collecting all serial sections (approximately 200–300 sections from each ovary). Therefore, we can safely say that the number of mice used for in vivo experiments was not too small to draw a conclusion.

Thus, we have demonstrated that mangafodipir confers pharmacological protection on the ovaries during chemotherapy with cisplatin and paclitaxel partially via the downregulation of ROS, without interfering with the antitumor activity of these agents in mice. Mangafodipir, therefore, though its efficacy might be limited, may represent a new alternative for the preservation of fertility in female patients with cancer, particularly as it is already approved for human administration. However, the specific mechanisms of oxidative stress leading to the activation of primordial follicles still need to be discussed more extensively. At the same time, further experiments will be needed to verify the existence of other potential mechanisms of mangafodipir on inhibition the activation of primordial follicles.

## Conclusions

In conclusion, mangafodipir exerts superoxide dismutase-mimetic activity, may partially attenuate the ovarian damage caused by cisplatin and paclitaxel without affecting their antitumor activity. Mangafodipir may therefore represent a new alternative for the preservation of fertility.

## Abbreviations

4-HNE: 4-hydroxynonenal
GCs: Granulosa cells
IHC: Immunohistochemistry
MnDPDP: Manganese dipyridoxyl diphosphate
ROS: Reactive oxygen species
SOD: Superoxide dismutase
